# The Relationship between Age, Gender, BMI, Diet, Salivary pH and Periodontal Pathogenic Bacteria in Children and Adolescents: A Cross-Sectional Study

**DOI:** 10.3390/biomedicines11092374

**Published:** 2023-08-24

**Authors:** Georgiana Veronica Motoc, Raluca Iulia Juncar, Abel Emanuel Moca, Ovidiu Motoc, Luminița Ligia Vaida, Mihai Juncar

**Affiliations:** 1 Doctoral School of Biomedical Sciences, University of Oradea, 1 Universității Street, 410087 Oradea, Romania; veromotoc@yahoo.com; 2 Department of Dentistry, Faculty of Medicine and Pharmacy, University of Oradea, 10 Piața 1 Decembrie Street, 410073 Oradea, Romania; ovidiumotoc3@gmail.com (O.M.); ligia_vaida@yahoo.com (L.L.V.); mihaijuncar@gmail.com (M.J.)

**Keywords:** oral microbiome, children, adolescents, Romania

## Abstract

The oral microbiome can be influenced by many factors and its dysbiosis can have negative effects on oral and general health. The purpose of this study was to analyze the intensity of 11 periodontal pathogenic microorganisms identified in the oral cavity of a sample of children and adolescents from Oradea, Romania and to investigate the association of some variables (age, gender, body mass index, diet, and salivary pH) with the identified microorganisms. The cross-sectional study was conducted on a group of clinically healthy patients under the age of 18 years from Oradea, Romania. For the analysis of the periodontal pathogens, the micro-IDent kit was used, which determines 11 bacterial markers for periodontitis and peri-implantitis. The kit is based on the polymerase chain reaction (PCR). Bacterial sampling was carried out according to the manufacturer’s instructions. A total of 60 children (23 male, 37 female) were included in this study, and were divided into three different age categories. No statistically significant results were identified for gender. However statistically significant results were obtained for other variables. Positive results for *Prevotella intermedia* and *Bacteroides forsythus* were associated with ages between 13 and 18 years, while positive results for *Capnocytophaga* spp. were associated with ages between 2 and 5 years. Positive results for *Prevotella intermedia*, *Bacteroides forsythus*, *Peptostreptococcus micros*, *Campylobacter rectus* and *Eikenella corodens* were associated with an overweight BMI. Negative results for *Prevotella intermedia* and *Eikenella corodens* were associated with a natural diet in the first 6 months of life. Positive results for *Fusobacterium nucleatum* and *Campylobacter rectus* were associated with an acidic salivary pH. In this study, the identified periodontal pathogens were associated with age, body mass index, diet in the first 6 months of life, and salivary pH.

## 1. Introduction

The oral microbiome is represented by the collective genome of microorganisms residing in the oral cavity and is composed of a core microbiome, which is common to all individuals, and a variable microbiome, which is unique to each individual [1]. The oral microbiome consists of over 1000 bacterial species from different microbial phyla, such as *Actinobacteria*, *Bacteroidetes*, *Chlamydia*, *Euryarchaeota*, *Fusobacteria*, *Firmicutes*, *Proteobacteria*, *Spriochaetes* and *Tenericutes* [2]. Of these only 54% have been cultivated and identified [3]. Apart from the bacteria that dominate the biomass of the oral habitat, fungal genera such as *Candida*, *Aspergillus*, and *Gibberella* have also been identified [4]. Maintaining the balance of the oral microbiome impacts oral and general health [5] and has an active role in the physiological, nutritional, and defense development of each individual [3].

The variable component of the oral microbiome ensures a microbial identity for each individual [6], and its development begins immediately after birth and with the first feeding of the child [1]. The oral microbiome of the child rapidly becomes more diverse and complex [7]. The first two years of life represent an essential period for the development of the oral microbiome [8]. The pioneer bacteria colonize the oral cavity immediately after birth and are followed by other colonizing bacteria, generally facultative anaerobic, such as *Streptococcus* and *Neisseria*, and obligate anaerobic like *Lactobacillus*, *Actinomyces*, and *Veillonella* [9].

The homeostasis of the oral microbial community can be influenced by environmental and community factors, but also by interactions between different body organs. Vaccines, passive smoking, or antibiotics impact this homeostasis [10]. It seems that diet influences the composition of the oral microbiome, and breastfeeding for no more than 12 months contributes to the formation of a healthier microbiome [6] and has a protective role against dental caries [11].

Disturbances in the homeostasis of the oral microbiome can lead to the development of pathologies with oral tropism or can influence the development of general diseases [4]. Periodontal diseases, dental caries, oral cancer, and viral diseases with oral manifestations have been associated with different oral microorganisms [4]. In addition, the microbiome of the oral cavity has been associated with the development of inflammatory bowel disease (IBD), liver diseases, cardiovascular diseases, diabetes, rheumatoid arthritis, and various types of cancer [12]. Esophageal, pancreatic, and colorectal cancer can be related to different oral dysbiosis [12].

Periodontal disease can manifest as an inflammation of the gingiva, bone, and ligaments supporting the tooth [13]. It mainly affects adults [14] but it can also affect children, and periodontal pathogens can be present in the oral microbiome from a young age [15]. Pathogenic periodontal bacteria are numerous [16] and, for their identification, test kits are available. They are easy to use in both children and adults [17].

Despite the numerous studies that have aimed to research the human oral microbiome, information on the development of the oral microbiome in children is limited and incomplete [18]. The factors that influence the development of the microbiome still need to be researched, as well [2]. Moreover, it seems that there is also a geographical influence on the development of the oral microbiome and studies in different geographical regions are needed to identify the preferential composition of the microbiome in populations from different parts of the world [19]. Periodontal pathogenic bacteria are not sufficiently studied in children and, in Romania, there are no studies that investigated the oral periodontal pathogenic bacteria in children and adolescents. Until the design of this manuscript, the authors could not identify any article that dealt with this subject.

Considering the previously stated aspects, the purpose of this study was to investigate the intensity of 11 periodontal pathogenic microorganisms identified in the oral microbiome of a sample of children and adolescents from Oradea, Romania. Another aim was to analyze the association between the investigated variables (such as age, gender, body mass index, diet, and salivary pH) and the periodontal pathogenic bacteria in the studied sample.

## 2. Materials and Methods

### 2.1. Ethical Considerations

The study was conducted according to the principles formulated in the 2008 Declaration of Helsinki and its subsequent amendments. For participation in this study, written consent was requested from the parents or legal guardians of children under the age of 18. They were not charged any additional fee for analyzing the periodontal pathogenic bacteria and were not promised any financial benefit. The study was approved by the Research Ethics Committee of the Faculty of Medicine and Pharmacy of the University of Oradea (IRB No. CEFMF/10 from 30 May 2022).

### 2.2. Study Design and Participants

The research was designed as a cross-sectional study and was carried out on a sample of children and adolescents under the age of 18 from Oradea, Romania. The specimens were collected from individuals in a private dental office in Oradea, Romania between October 2021 and April 2022. The study sample included clinically healthy children (with no systemic diseases), who did not have any ongoing treatment for an acute or chronic disease, who lived in Romania and whose parents agreed to include the children in this study.

### 2.3. Investigation of the Oral Microbiome

To investigate the periodontal pathogens, the micro-IDent test kit (Hain Lifescience GmbH, Nehren, Germany) was used. The test kit uses the polymerase chain reaction (PCR) technique. The test determines 11 bacterial markers for periodontitis and peri-implantitis: *Actinobacillus actinomycetemcomitans*, *Porphyromonas gingivalis*, *Prevotella intermedia*, *Bacteroides forsythus*, *Treponema denticola*, *Peptostreptococcus micros*, *Fusobacterium nucleatum*/*periodonticum*, *Eikenella corrodens*, *Campylobacter rectus*, *Eubacterium nodatum*, and *Capnocytophaga* spp. [20]. The bacterial sampling was carried out by one operator, in the morning, before brushing. The sample originated from the gingival crevicular fluid and was collected with the help of the paper sticks included in the test kit. The sticks were inserted into the sulcus of the investigated teeth, as specified by the manufacturer [20], and were left in place for 10 s. After the 10 s, they were withdrawn and inserted into transfer tubes. The tube was placed in a box together with a sheet containing the child’s data. The sample was transported to a medical analysis laboratory, having a stability of 7 days at temperatures of 2–8 °C.

The samples were placed in individual 1.5 mL containers, to which 150 mL of 5% Chelex solution was added. The tubes containing the samples were then centrifuged and then vortexed, after which they were heated to a temperature of 95 °C. For the PCR test, 5 mL of the supernatant solution was used. The samples were amplified in a 50 μL reaction volume consisting of 5.0 μL of template DNA and 45 μL of reaction mixture containing 35 μL of primer-nucleotide-PNM (Micro-IDent) mixture, 5.0 μL of 10× PCR buffer, 5.0 μL of 2.5 mM MgCl2, and 0.2 U Taq at the hot start (Qiagen, GmbH, Hilden, Germany). PCR cycling was performed in GTQ-Cycler 96 thermal cycler (HAIN Lifescience, Nehren, Germany). Negative control was included in the test sample. Negative control was 5.0 μL of sterile water PCR, each added to 45 μL of the reaction mixture. Subsequently, the reverse hybridization was performed according to the Micro-IDent^®^plus test (HAIN Lifescience GmbH, Nehren, Germany). Reverse hybridization subsequent reverse hybridization was performed according to the Micro-IDent^®^plus. Briefly, amplicon biotinylate was denatured and incubated in Twincubator^®^ (HAIN Lifescience, Nehren, Germany) at 45 °C with hybridization buffer and coated strips with two control lines (conjugates and amplification) with six (Micro-IDent^®^plus) species-specific probes [21]. Following the binding of the PCR products to the corresponding probe complements, any nonspecific bound DNA was eliminated using a very precise washing procedure. Alkaline phosphatase conjugated to streptavidin was added, the samples were cleaned, and an alkaline phosphatase substrate was added to enable visualization of the hybridization results [22]. The micro-Ident test allows for the detection of 11 periodontal pathogens with a detection limit of 10 × 10^3^ bacterial counts for *A. actinomycetemcomitans* and 10 × 10^4^ for the remaining pathogens. Strips were used to determine the intensity of the identified microorganisms. They were scanned and the bands related to the pathogens on the strips were visualized on a computer. They were analyzed by measuring the contrast luminescence amounts of the bands. A clear band meant that the results were negative. An invisible-looking band meant that the result was weak positive. A weak-looking band meant that the result was positive. A normal-looking band meant that the result was intense positive [23].

The Saliva Check Buffer test (GC, Tokyo, Japan) was used to test the salivary pH. In order to test the pH of the resting saliva, a collection cup, a pH strip, and a pH indicator are required, all included in the test. To check the pH of the resting saliva, the child was asked to expectorate into the collection cup. A pH strip was inserted into the expectorated saliva for 10 s. After the 10 s, the color of the strip was checked. Acidic saliva, with a pH below 6.6, would have had a chromatic counterpart in the red or yellow section of the indicator, pH-neutral and alkaline saliva, with a pH of 6.7 or more, would have had a chromatic counterpart in the green section of the indicator [24]. The salivary pH was checked by the same operator, during the same appointment.

Body mass index (BMI) was calculated by dividing weight (expressed in kilograms) by height squared (expressed in meters), by using the formula BMI = W (kg)/H^2^ (m^2^) [25].

The following variables were investigated: age (2–5 years, 6–12 years, 13–18 years), gender (male, female), body mass index or BMI (underweight, normal, overweight), salivary pH (neutral, acidic, alkaline), and the type of diet up to 6 months of life (natural—breastfeeding, mixed—breastfeeding and formula, artificial—formula).

### 2.4. Statistical Analysis

Statistical analysis was performed using IBM SPSS Statistics 26 (IBM, Chicago, IL, USA) and Microsoft Office Excel/Word 2013 (Microsoft, Redmond, DC, USA). Categorical variables were expressed in absolute form or percentages and were tested by using Fisher’s exact test. Z-tests with Bonferroni correction were performed in order to detail the results after obtaining the contingency tables.

## 3. Results

The data in Table 1 show the characteristics of the children investigated in the study. A total of 60 individuals were included in the study sample. There were more female subjects than male subjects (61.7% vs. 38.3%). Most children were aged between 6 and 12 years (60.0%), and of normal weight (75.0%). Their diets were predominantly natural (55.0%) or mixed (35.0%). The measured salivary pH was predominantly neutral (48.3%).

**Figure 1:**
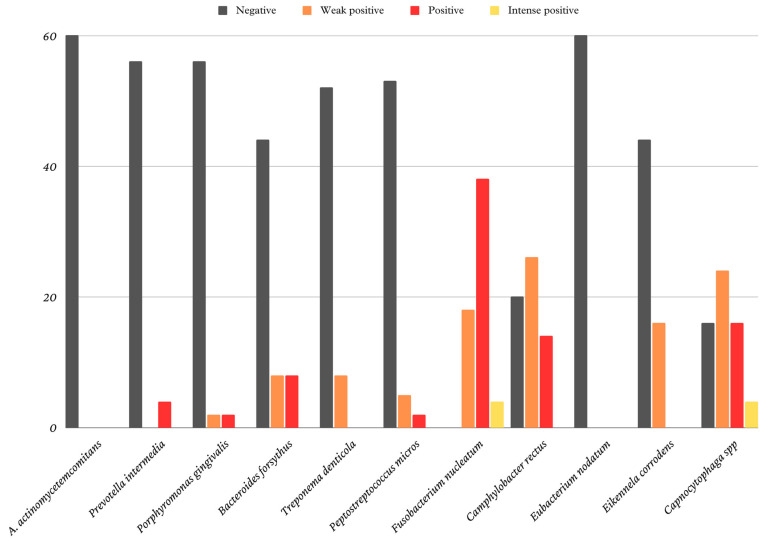
Identified microorganisms and their prevalence in the samples.

Various bacterial species have been identified in the oral microbiome. The identified bacterial species and their prevalence in the collected samples are shown in Figure 1.

The data in Table 2 represent the distribution of children by gender, age, BMI, and the intensity of the microorganisms identified. Gender had no statistically significant influence on any of the microorganisms identified, according to the Fisher test.

Age had some statistically significant influences on some of the microorganisms. For *P. intermedia* (*p* < 0.001), *B. forsythus* (*p* < 0.001), *T. denticola* (*p* = 0.005), and *Capnocytophaga* spp. (*p* = 0.001) the differences between the groups were observed to be statistically significant according to the Fisher test and the Z tests with Bonferroni correction showed that:

- Negative results for *P. intermedia* were associated mainly with ages between 2 and 5 years (28.6%) and 6 and 12 years (64.3%), while positive results were associated only with ages between 13 and 18 years (100%);
- Weak positive results for *B. forsythus* were associated mainly with ages between 6 and 12 years (87.5%), while positive results were associated mainly with ages between 13 and 18 years (75.0%);
- For *T. denticola*, weak positive results were associated with ages between 6 and 12 years (50.0%) or 13 and 18 years (50.0%);
- For *Capnocytophaga* spp., positive results were associated with ages between 2 and 5 years (56.3%) and 6 and 12 years (43.8%), while negative results were associated with ages between 6 and 12 years (62.5%) and 13 and 18 years (37.5%).

As for BMI, significant results were identified regarding the distribution of children according to BMI and *P. intermedia* (*p* = 0.021), *B. forsythus* (*p* = 0.005), *P. micros* (*p* < 0.001), *C. rectus* (*p*= 0.004), and *E. corrodens* (*p* = 0.003). Z-tests with Bonferroni correction showed that:

- For *P. intermedia*, positive results were associated with a normal BMI (50.0%) or an overweight BMI (50.0%);
- For *B. forsythus*, weak positive or positive results were mainly associated with a normal BMI (50.0% in both groups);
- Weak positive results for *P. micros*, were mainly associated with an underweight BMI (80.0%), negative results were mainly associated with a normal BMI (83.0%), while positive results were only associated with an overweight BMI (100%);
- Positive results for *C. rectus* were associated with a normal BMI (71.4%) or an overweight BMI (28.6%);
- For *E. corrodens*, weak positive results were mainly associated with a normal BMI (68.8%) or an overweight BMI (25.0%).

The data in Table 3 represent the distribution of children by diet, salivary pH and the intensity of the microorganisms identified in the collected samples. For *P. intermedia* (*p* = 0.021) and for *E. corodens* (*p* = 0.022), the differences between the groups were observed to be statistically significant according to the Fisher test and the Z tests with Bonferroni correction show that:

- For *P. intermedia*, negative results were mainly associated with a natural diet (58.9%), while positive results were only associated with a mixed diet (100%);
- Negative results for *E. corodens* were mainly associated with a natural diet (63.6%), while weak positive results were mainly associated with a mixed diet (43.8%).

According to the Fisher test, statistically significant results were identified regarding the distribution of children according to salivary pH and *T. denticola* (*p* = 0.006), *F. nucleatum* (*p* = 0.010), *C. rectus* (*p* = 0.023), and *Capnocytophaga* spp. (*p* =0.002). Z-tests with Bonferroni correction showed that:

- For *T. denticola*, weak positive results were only associated with a neutral salivary pH (100%);
- For *F. nucleatum*, weak positive results were mainly associated with a neutral (38.9%) or an acidic salivary pH (38.9%), and intense positive results were associated mainly with an acidic salivary pH (75.0%);
- Negative results for *C. rectus* were mainly associated with a neutral salivary pH (60.0%), while positive results were mainly associated with an acidic (50.0%) or an alkaline (35.7%) salivary pH;
- For *Capnocytophaga* spp., negative results were mainly associated with a neutral salivary pH (87.5%), while intense positive results were mainly associated with an alkaline salivary pH (50.0%).

## 4. Discussion

Most microorganisms that form the oral microbiome constitute the oral biofilm [26], the different genera of bacteria having a preferential distribution for three distinct habitat areas, i.e., for dental plaque, the lingual dorsum, and the keratinized gingiva [27]; at the same time populating other areas of the oral cavity [28]. Under normal circumstances, the microbiome of the oral cavity maintains a symbiotic balance known as eubiosis [27]. When changes occur in the normal structure of the microbiome, dysbioses appears, which if left unaddressed can lead to the appearance of oral or general pathologies [27]. Considering that variation in the oral microbiome was identified depending on the geographical location of the individuals [19,29], we wanted to highlight the presence or absence of certain microorganisms in a sample of children and adolescents from Oradea, Romania. The oral microbiome seems to be influenced by multiple factors [30], so in this study we followed the influence of variables such as age, gender, or body mass index on the investigated microorganisms.

In this study, the micro-IDent test kit (Hain Lifescience GmbH, Nehren, Germany) was used, and it is based on the identification of bacteria by the polymerase chain reaction (PCR) technique [20]. Although there are other diagnostic tests available on the market, micro-IDent is available in Romania and has a high specificity, correctly identifying 11 types of bacteria [31], being preferred in this study due to these reasons. Among the bacteria identified by this test, *A. actinomycetemcomitans* is an anaerobic gram-negative microorganism with a proven role in the occurrence of periodontal disease [32], as are *P. gingivalis* [33], and the gram-negative bacterium *T. forsythia* [34]. *T. denticola* has been identified both as a pathogen in periodontal disease, but also in other oral diseases, as well as periapical pathologies, peri-implant diseases, and oral tumors [35]. *P. micros*, *F. nucleatum*, *C. rectus*, and *F. nodatum* are other identified bacteria that have a role in the establishment and evolution of periodontal disease [36,37]. Moreover, it seems that *F. nucleatum* could act as an oncogenic agent and lead to the appearance of colorectal cancer [38]. Besides these, the test also identifies the presence of *E. corrodens* and *Capnocytophaga* spp. [20].

One of the variables that was investigated in this study was age and the influence of age on the identified microorganisms. The children were divided into three age groups (2–5 years, 6–12 years, 13–18 years) because these age categories correspond to the 3 major developmental stages (preschool, school and adolescence) [39]. Positive or, at least weak positive, result for *P. intermedia*, *B. forsythus* and *T. denticola* were associated with age between 13 and 18 years, while positive results for *Capnocytophaga* spp. were associated with age between 2 and 5 years. Statistically significant results related to age and only to these four microorganisms were identified. *P. intermedia*, *B. forsythus*, and *T. denticola* are bacteria that can cause periodontal diseases [34,35,40]. The most common periodontal disease in children and adolescents is gingivitis, children over 12 years of age being more affected by periodontal diseases [41], just like in this study. The presence of these bacteria indicates the existence of a degree of gingival inflammation, more common in adolescents due to pubertal gingivitis [42], but also due to poor oral hygiene [43]. Gender had no statistically significant influence on the evaluated microorganisms in this study. If this is true for children and adolescents, studies conducted on adult populations show different results. Zhao et al. (2021) identified differences between male and female patients in the composition of the oral microbiome of elderly patients [44], and so have Liu et al. [45], and Han and Park (2018) [46]. This aspect may be due to the fact that the microbiome of the oral cavity evolves throughout life, the composition being strongly influenced by lifestyle habits, which act in the long term [47].

Body mass index is used to identify people with weight and obesity issues and can be used in both children and adults [48]. This variable was taken into account because there is an association between obesity and an increased prevalence of dental caries [49,50]. Moreover, there is an association between obesity and a higher prevalence of periodontal disease [51]. Both dental caries and periodontal disease appear as a result of a dysbiosis at the level of the oral microbiome, with the installment of some changes in the normal bacterial population [27]. In this study, statistically significant results were identified in the distribution of children according to BMI and five microorganisms (*P. intermedia*, *B. forsythus*, *P. micros*, *C. rectus*, and *E. corrodens*). For all these microorganisms, an overweight BMI was associated with positive or weak positive results of these pathogens. Similar to these results, Maciel et al. (2016) obtained associations of obesity with high levels of periodontal pathogens [52] and overweight patients seem to have higher levels of *T. forsythia* and more severe forms of periodontal disease [53]. The results are similar in the pediatric population [54].

The influence of diet on the oral microbiome has been studied for a long time, results suggesting that diet plays an important role in the occurrence of dysbioses that lead to the appearance of various oral pathologies [55]. It seems that the development of the oral microbiome begins in utero and until the 5th month of life, a large part of the microbiome of the oral cavity comes from breast milk, in breastfed children [56]. It seems that there is a difference between children that are naturally fed (breastfed) and those that are artificially fed (formula) in the first 6 months of life, different bacterial species dominating in children fed naturally, mixed, or artificially [56]. In the current study, statistically significant results were obtained for *P. intermedia* and *E. corodens*. Negative results for *P. intermedia* and *E. corodens* were mainly associated with a natural diet in the first 6 months of life. *Prevotella* is a microorganism that predominates in the oral cavity immediately after birth, in naturally born children [56]. Positive results of *P. intermedia* were mainly associated with a mixed diet, consisting of a mixture of formula and breast milk. For salivary pH, statistically significant results were identified for *T. denticola*, *F. nucleatum*, *C. rectus*, *Capnocytophaga* spp., and these results were expected, considering that the important role of salivary pH in the occurrence of dysbiosis in the oral cavity is known [57].

This study offers an in-depth look at the periodontal pathogenic bacteria of the children and adolescents included in the studied sample and contains valuable information regarding the influence of the various investigated variables on the investigated microorganisms. Although we consider it a valuable study, there are some limitations that we have identified. A limitation would be related to the total number of children from the studied sample. It is possible to obtain more statistically significant results on a larger sample of children. Also, in this study we investigated the bacteria that have a role in periodontal disease and not in the occurrence of dental caries, considering that these were the bacteria that could be identified by the diagnostic kit that was used. We only looked at the 11 periodontic pathogenic bacteria that were described in Section 2. We also investigated the role of the type of diet carried out up to 6 months of life and it is possible that mothers wrongly reported the type and duration of the child’s natural, artificial, or mixed feeding.

## 5. Conclusions

In this study, statistically significant results were identified for some of the microorganisms and the investigated variables. The only variable that did not have a statistically significant association with the periodontal pathogenic bacteria was the gender of the children. Positive results for *P. intermedia* and *B. forsythus* were mainly associated with the 13–18 years age group. Positive results for *P. intermedia*, *B. forsythus*, *P. micros*, and *C. rectus* were mainly associated with an overweight BMI, and negative results for *P. intermedia* and *E. corrodens* were mainly associated with a natural diet in the first 6 months of life. The periodontal pathogenic bacteria were therefore influenced by age, BMI, diet in the first 6 months of life, and salivary pH. The investigation of some additional variables (s.a. periodontal status, DMFT index) is recommended to create an even more complex picture of the studied sample of children. Developing and implementing educational programs regarding oral health aimed at parents would be useful for the control of the identified microorganisms, and the subsequent diseases.

## Figures and Tables

**Table 1:** Sample characteristics.

Variable		No.	%
**Gender**	Male	23	38.3%
	Female	37	61.7%
**Age group**	0–5 years	16	26.7%
	6–12 years	36	60.0%
	13–18 years	8	13.3%
**BMI**	Underweight	11	18.3%
	Normal	45	75.0%
	Overweight	4	6.7%
**Diet**	Natural	33	55.0%
	Mixed	21	35.0%
	Artificial	6	10.0%
**Salivary pH**	Neutral	29	48.4%
	Acidic	14	23.3%
	Alkaline	17	28.3%

BMI, body mass index; No., number; %, percentage.

**Table 2:** Distribution according to gender, age, BMI, and identified microorganisms.

	*P. intermedia*	*P. gingivalis*	*B. forsythus*	*T. denticola*	*P. micros*	*F. nucleatum*	*C. rectus*	*E. corrodens*	*Capnocytophaga* spp.
Gender	Negative								
Male	21 (37.5%)	21 (37.5%)	17 (38.6%)	20 (38.5%)	21 (39.6%)	0 (0.0%)	8 (40.0%)	17 (39.6%)	6 (37.5%)
Female	35 (62.5%)	35 (62.5%)	27 (61.4%)	32 (61.5%)	32 (60.4%)	0 (0.0%)	12 (60.0%)	27 (61.4%)	10 (62.5%)
	Weak positive								
Male	0 (0.0%)	1 (50.0%)	3 (37.5%)	3 (37.5%)	2 (40.0%)	7 (38.9%)	10 (38.5%)	6 (37.5%)	9 (37.5%)
Female	0 (0.0%)	1 (50.0%)	5 (62.5%)	5 (62.5%)	3 (60.0%)	11 (61.1%)	16 (61.5%)	10 (62.5%)	15 (62.5%)
	Positive								
Male	2 (50.0%)	1 (50.0%)	3 (37.5%)	0 (0.0%)	0 (0.0%)	16 (42.1%)	5 (35.7%)	0 (0.0%)	5 (31.3%)
Female	2 (50.0%)	1 (50.0%)	5 (62.5%)	0 (0.0%)	2 (100%)	22 (57.9%)	9 (64.3%)	0 (0.0%)	11 (68.8%)
	Intense positive								
Male	0 (0.0%)	0 (0.0%)	0 (0.0%)	0 (0.0%)	0 (0.0%)	0 (0.0%)	0 (0.0%)	0 (0.0%)	3 (75.0%)
Female	0 (0.0%)	0 (0.0%)	0 (0.0%)	0 (0.0%)	0 (0.0%)	4 (100%)	0 (0.0%)	0 (0.0%)	1 (25.0%)
*p* *	0.634	1.000	1.000	1.000	0.827	0.359	1.000	1.000	0.495
Age	Negative								
2–5 years	16 (28.6%)	16 (28.6%)	15 (34.1%)	16 (30.8%)	15 (28.3%)	0 (0.0%)	2 (10.0%)	12 (27.3%)	0 (0.0%)
6–12 years	36 (64.3%)	32 (57.1%)	27 (61.4%)	32 (61.5%)	30 (56.6%)	0 (0.0%)	16 (80.0%)	28 (63.6%)	10 (62.5%)
13–18 years	4 (7.1%)	8 (14.3%)	2 (4.5%)	4 (7.7%)	8 (15.1%)	0 (0.0%)	2 (10.0%)	4 (9.1%)	6 (37.5%)
	Weak positive								
2–5 years	0 (0.0%)	0 (0.0%)	1 (12.5%)	0 (0.0%)	0 (0.0%)	5 (27.8%)	7 (26.9%)	4 (25.0%)	7 (29.2%)
6–12 years	0 (0.0%)	2 (100%)	7 (87.5%)	4 (50.0%)	5 (100%)	11 (61.1%)	15 (57.7%)	8 (50.0%)	15 (62.5%)
13–18 years	0 (0.0%)	0 (0.0%)	0 (0.0%)	4 (50.0%)	0 (0.0%)	2 (11.1%)	4 (15.4%)	4 (25.05)	2 (8.3%)
	Positive								
2–5 years	0 (0.0%)	0 (0.0%)	0 (0.0%)	0 (0.0%)	1 (50.0%)	10 (26.3%)	7 (50%)	0 (0.0%)	9 (56.3%)
6–12 years	0 (0.0%)	2 (100%)	2 (25.0%)	0 (0.0%)	1 (50.0%)	22 (57.9%)	5 (35.7%)	0 (0.0%)	7 (43.8%)
13–18 years	4 (100%)	0 (0.0%)	6 (75.0%)	0 (0.0%)	0 (0.0%)	6 (15.85)	2 (14.3%)	0 (0.0%)	0 (0.0%)
	Intense positive								
2–5 years	0 (0.0%)	0 (0.0%)	0 (0.0%)	0 (0.0%)	0 (0.0%)	1 (25.0%)	0 (0.0%)	0 (0.0%)	0 (0.0%)
6–12 years	0 (0.0%)	0 (0.0%)	0 (0.0%)	0 (0.0%)	0 (0.0%)	3 (75.0%)	0 (0.0%)	0 (0.0%)	4 (100%)
13–18 years	0 (0.0%)	0 (0.0%)	0 (0.0%)	0 (0.0%)	0 (0.0%)	0 (0.0%)	0 (0.0%)	0 (0.0%)	0 (0.0%)
*p* *	<0.001	1.000	<0.001	0.005	0.423	1.000	0.087	0.311	0.001
BMI	Negative								
Underweight	11 (19.6%)	9 (16.1%)	7 (15.9%)	7 (13.5%)	7 (13.2%)	0 (0.0%)	6 (30.0%)	10 (22.7%)	6 (37.5%)
Normal	43 (76.8%)	43 (76.8%)	37 (84.1%)	41 (78.8%)	44 (83%)	0 (0.0%)	14 (70.0%)	34 (77.3%)	10 (62.5%)
Overweight	2 (3.6%)	4 (7.1%)	0 (0.0%)	4 (7.7%)	2 (3.8%)	0 (0.0%)	0 (0.0%)	0 (0.0%)	0 (0.0%)
	Weak positive								
Underweight	0 (0.0%)	0 (0.0%)	2 (25.0%)	4 (50.0%)	4 (80.0%)	2 (11.1%)	5 (19.2%)	1 (6.3%)	2 (8.3%)
Normal	0 (0.0%)	2 (100%)	4 (50.0%)	4 (50.0%)	1 (20.0%)	14 (77.8%)	21 (80.8%)	11 (68.8%)	19 (79.2%)
Overweight	0 (0.0%)	0 (0.0%)	2 (25.0%)	0 (0.0%)	0 (0.0%)	2 (11.1%)	0 (0.0%)	4 (25.0%)	3 (12.5%)
	Positive								
Underweight	0 (0.0%)	2 (100%)	2 (25.0%)	0 (0.0%)	0 (0.0%)	9 (23.7%)	0 (0.0%)	0 (0.0%)	3 (18.8%)
Normal	2 (50.0%)	0 (0.0%)	4 (50.0%)	0 (0.0%)	0 (0.0%)	27 (71.1%)	10 (71.4%)	0 (0.0%)	12 (75.0%)
Overweight	2 (50.0%)	0 (0.0%)	2 (25.0%)	0 (0.0%)	2 (100%)	2 (5.3%)	4 (28.6%)	0 (0.0%)	1 (6.2%)
	Intense positive								
Underweight	0 (0.0%)	0 (0.0%)	0 (0.0%)	0 (0.0%)	0 (0.0%)	0 (0.0%)	0 (0.0%)	0 (0.0%)	0 (0.0%)
Normal	0 (0.0%)	0 (0.0%)	0 (0.0%)	0 (0.0%)	0 (0.0%)	4 (100%)	0 (0.0%)	0 (0.0%)	4 (100%)
Overweight	0 (0.0%)	0 (0.0%)	0 (0.0%)	0 (0.0%)	0 (0.0%)	0 (0.0%)	0 (0.0%)	0 (0.0%)	0 (0.0%)
*p* *	0.021	0.124	0.005	0.081	<0.001	0.642	0.004	0.003	0.242

* Fisher’s Exact Test.

**Table 3:** Distribution according to diet, salivary pH, and identified microorganisms.

	*P. intermedia*	*P. gingivalis*	*B. forsythus*	*T. denticola*	*P. micros*	*F. nucleatum*	*C. rectus*	*E. corrodens*	*Capnocytophaga* spp.
Diet	Negative								
Natural	33 (58.9%)	29 (51.85)	27 (61.4%)	31 (59.6%)	29 (54.7%)	0 (0.0%)	14 (70.0%)	28 (63.6%)	10 (62.5%)
Mixed	17 (30.4%)	21 (37.5%)	12 (27.3%)	15 (28.8%)	20 (37.7%)	0 (0.0%)	6 (30.0%)	14 (31.8%)	6 (37.5%)
Artificial	6 (10.7%)	6 (10.7%)	5 (11.3%)	6 (11.5%)	4 (7.5%)	0 (0.0%)	0 (0.0%)	2 (4.6%)	0 (0.0%)
	Weak positive								
Natural	0 (0.0%)	2 (100%)	4 (50.0%)	2 (25.0%)	4 (80.0%)	7 (38.9%)	14 (53.8%)	5 (31.2%)	16 (66.7%)
Mixed	0 (0.0%)	0 (0.0%)	3 (37.5%)	6 (75.0%)	0 (0.0%)	6 (33.3%)	9 (34.6%)	7 (43.8%)	6 (25.0%)
Artificial	0 (0.0%)	0 (0.0%)	1 (12.5%)	0 (0.0%)	1 (20.0%)	5 (27.8%)	3 (11.6%)	4 (25.0%)	2 (8.3%)
	Positive								
Natural	0 (0.0%)	2 (100%)	2 (25.0%)	0 (0.0%)	0 (0.0%)	23 (60.5%)	5 (35.7%)	0 (0.0%)	7 (43.8%)
Mixed	4 (100%)	0 (0.0%)	6 (75.0%)	0 (0.0%)	1 (50.0%)	14 (36.8%)	6 (42.9%)	0 (0.0%)	7 (43.8%)
Artificial	0 (0.0%)	0 (0.0%)	0 (0.0%)	0 (0.0%)	1 (50.0%)	1 (2.7%)	3 (21.4%)	0 (0.0%)	2 (12.4%)
	Intense positive								
Natural	0 (0.0%)	0 (0.0%)	0 (0.0%)	0 (0.0%)	0 (0.0%)	3 (75.0%)	0 (0.0%)	0 (0.0%)	0 (0.0%)
Mixed	0 (0.0%)	0 (0.0%)	0 (0.0%)	0 (0.0%)	0 (0.0%)	1 (25.0%)	0 (0.0%)	0 (0.0%)	2 (50.0%)
Artificial	0 (0.0%)	0 (0.0%)	0 (0.0%)	0 (0.0%)	0 (0.0%)	0 (0.0%)	0 (0.0%)	0 (0.0%)	2 (50.0%)
*p* *	0.021	0.613	0.157	0.070	0.077	0.059	0.180	0.022	0.055
Salivary pH	Negative								
Neutral	27 (48.2%)	25 (44.6%)	17 (38.6%)	21 (40.4%)	25 (47.2%)	0 (0.0%)	12 (60.0%)	21 (47.7%)	14 (87.5%)
Acidic	14 (25.0%)	14 (25.0%)	12 (27.3%)	14 (26.9%)	12 (22.6%)	0 (0.0%)	4 (20.0%)	10 (22.7%)	2 (12.5%)
Alkaline	15 (26.8%)	17 (30.4%)	15 (34.1%)	17 (32.7%)	16 (30.2%)	0 (0.0%)	4 (20.0%)	13 (29.6%)	0 (0.0%)
	Weak positive								
Neutral	0 (0.0%)	2 (100%)	6 (75.0%)	8 (100%)	4 (80.0%)	7 (38.9%)	15 (57.7%)	8 (50.0%)	10 (41.7%)
Acidic	0 (0.0%)	0 (0.0%)	2 (25.0%)	0 (0.0%)	0 (0.0%)	7 (38.9%)	3 (11.5%)	4 (25.0%)	4 (16.6%)
Alkaline	0 (0.0%)	0 (0.0%)	0 (0.0%)	0 (0.0%)	1 (20.0%)	4 (22.2%)	8 (30.8%)	4 (25.0%)	10 (41.7%)
	Positive								
Neutral	2 (50.0%)	2 (100%)	6 (75.0%)	0 (0.0%)	0 (0.0%)	22 (57.9%)	2 (14.3%)	0 (0.0%)	4 (25.0%)
Acidic	0 (0.0%)	0 (0.0%)	0 (0.0%)	0 (0.0%)	2 (100%)	4 (10.5%)	7 (50.0%)	0 (0.0%)	7 (43.8%)
Alkaline	2 (50.0%)	0 (0.0%)	2 (25.0%)	0 (0.0%)	0 (0.0%)	12 (31.6%)	5 (35.7%)	0 (0.0%)	5 (31.2%)
	Intense positive								
Neutral	0 (0.0%)	0 (0.0%)	0 (0.0%)	0 (0.0%)	0 (0.0%)	0 (0.0%)	0 (0.0%)	0 (0.0%)	1 (25.0%)
Acidic	0 (0.0%)	0 (0.0%)	0 (0.0%)	0 (0.0%)	0 (0.0%)	3 (75.0%)	0 (0.0%)	0 (0.0%)	1 (25.0%)
Alkaline	0 (0.0%)	0 (0.0%)	0 (0.0%)	0 (0.0%)	0 (0.0%)	1 (25.0%)	0 (0.0%)	0 (0.0%)	2 (50.0%)
*p* *	0.674	0.510	0.062	0.006	0.100	0.010	0.023	1.000	0.002

* Fisher’s Exact Test.

## Data Availability

The data presented in this study are available on request from the corresponding author. The data are not publicly available due to privacy reasons.
